# Natural Polysaccharide *β*-Glucan Protects against Doxorubicin-Induced Cardiotoxicity by Suppressing Oxidative Stress

**DOI:** 10.3390/nu14040906

**Published:** 2022-02-21

**Authors:** Xuan Wang, Yuting Ji, Dekui Jin, Jingyi Qi, Xuening Hou, Wenting Zhao, Shuaishuai Zhou, Chengying Zhang, Yongting Luo, Peng An, Junjie Luo

**Affiliations:** 1 Beijing Advanced Innovation Center for Food Nutrition and Human Health, College of Food Science and Nutritional Engineering, China Agricultural University, Beijing 100083, China; xuanwxuan@outlook.com; 2 Key Laboratory of Precision Nutrition and Food Quality, Department of Nutrition and Health, China Agricultural University, Beijing 100193, China; sy20213313322@cau.edu.cn (Y.J.); qipeiyan2992@163.com (J.Q.); hxn2580@163.com (X.H.); zhaowenting@cau.edu.cn (W.Z.); shuaishuaizhou@cau.edu.cn (S.Z.); 3 Department of General Practice, The Third Medical Center of Chinese PLA General Hospital, Beijing 100039, China; jindekui@163.com

**Keywords:** *β*-glucan, doxorubicin cardiotoxicity, heart failure, oxidative stress

## Abstract

Doxorubicin (DOXO) can be used to treat a variety of human tumors, but its clinical application is limited due to severe cardiotoxic side effect. Here, we explore the role of *β*-glucan in DOXO-induced cardiotoxicity in mice and study its underlying mechanism. When co-administered with DOXO, *β*-glucan was observed to prevent left ventricular dilation and fibrosis. In fact, DOXO reduces the activity of mitochondrial respiratory chain complex and enhances oxidative stress, which in turn impairs heart function. DOXO decreases the ATP production capacity of the heart and increases the ROS content, while *β*-glucan can restore the heart capacity and reduce oxidative stress. *β*-glucan also increases the activity of antioxidant enzymes GSH-PX and SOD, and reduces the level of MDA in the serum. In addition, the mRNAs of cardiac dysfunction marker genes *ANP*, *BNP* and *Myh7* were significantly increased after DOXO induction, however, they did not increase when combined with *β*-glucan administration. In conclusion, our results indicate that *β*-glucan can improve the antioxidant capacity of the heart, thereby serving as a potential therapeutic strategy to prevent DOXO-induced cardiotoxicity.

## 1. Introduction 

DOXO has been used for more than 50 years in the treatment of liver cancer, breast cancer and many other types of malignancies as an effective clinical anti-tumor drug. Especially in the later stage, the survival rate of patients treated with DOXO has been significantly improved. However, there are many side effects on the clinical application, such as dose-dependent acute or chronic cardiotoxicity, including irreversible degenerative cardiomyopathy and congestive heart failure [1]. The incidence of cardiotoxicity due to the use of DOXO is as high as 11% [2]. Despite its many negative effects, DOXO is still widely used because it is so effective in very frequent tumors such as breast cancer and no more suitable alternatives have been found.

DOXO-induced cardiotoxicity appears to be a multifactorial process. At present, the main strategies to prevent cardiotoxicity caused by DOXO are as follows: (1) Free radical scavengers, antioxidants and anti-inflammatory cytokines can reduce cardiotoxicity [3,4,5]. (2) Iron chelating agents can chelate iron in cells and prevent the production of iron-assisted oxidative free radicals, inhibit topoisomerase II to protect cardiomyocytes, such as dexrazoxane [6,7]. (3) Control the peak concentration of DOXO administration. It is generally believed that long-term exposure to moderate concentrations of DOXO will be safer than pulsed supply of higher concentrations of drugs [8]. (4) Use a drug delivery system. For example, liposomes or nanoparticles can preferentially target tumor tissues, thereby reducing the concentration of DOXO exposed to plasma [9]. Unfortunately, how to prevent and treat cardiotoxicity caused by DOXO has not yet been accepted clinically. Therefore, it is urgent to explore and study effective DOXO-induced cardiotoxic protective agents.

*β*-glucan is a polysaccharide of D-glucose monomers connected by *β*-glucan bonds, isolated from various natural sources, including yeast, mushrooms, bacteria, algae, barley and oats [10]. For more than half a century, the biological activity and clinical application of *β*-glucan have been extensively studied. (1) *β*-glucan stimulates immune homeostasis through *β*-glucan receptors present in the mucosal immune system and helps prevent diseases related to reduced immune function [11]. (2) *β*-glucan can reduce blood cholesterol and glucose concentration, thereby reducing the risk of diabetes, cardiovascular disease and diabetes [12]. (3) Soluble *β*-glucan will be fermented by microorganisms in the colon and converted into short-chain fatty acids with a variety of functions, such as immunomodulation [13] and mediation of colon cancer cells apoptosis [14] and prevention of obesity [15]. (4) *β*-glucan has anti-tumor activity, which is mainly mediated by enhancing tumor immunity and inducing the excretion of carcinogens in the intestine [16]. (5) It has been discovered that *β*-glucan can also trap free radicals and has anti-oxidation and free radical scavenger properties due to its polymerized structure. The antioxidant capacity is the most important mechanism proposed for the protective effect of *β*-glucan [17]. However, the effect of *β*-glucan on DOXO-induced cardiotoxicity and oxidative stress remains largely unknown. In this study, we aimed to evaluate whether *β*-glucan can prevent DOXO-induced cardiotoxicity by reducing oxidative stress and enhancing mitochondrial function.

## 2. Methods

### 2.1. Ethics Approval

All animal procedures were reviewed and approved by the Committee on the Ethics of Animal Experiments of China Agricultural University (Beijing, China), in accordance with the Guiding Principles for the Care and Use of Laboratory Animals.

### 2.2. DOXO Cardiotoxicity Protocols

**Figure 1:**
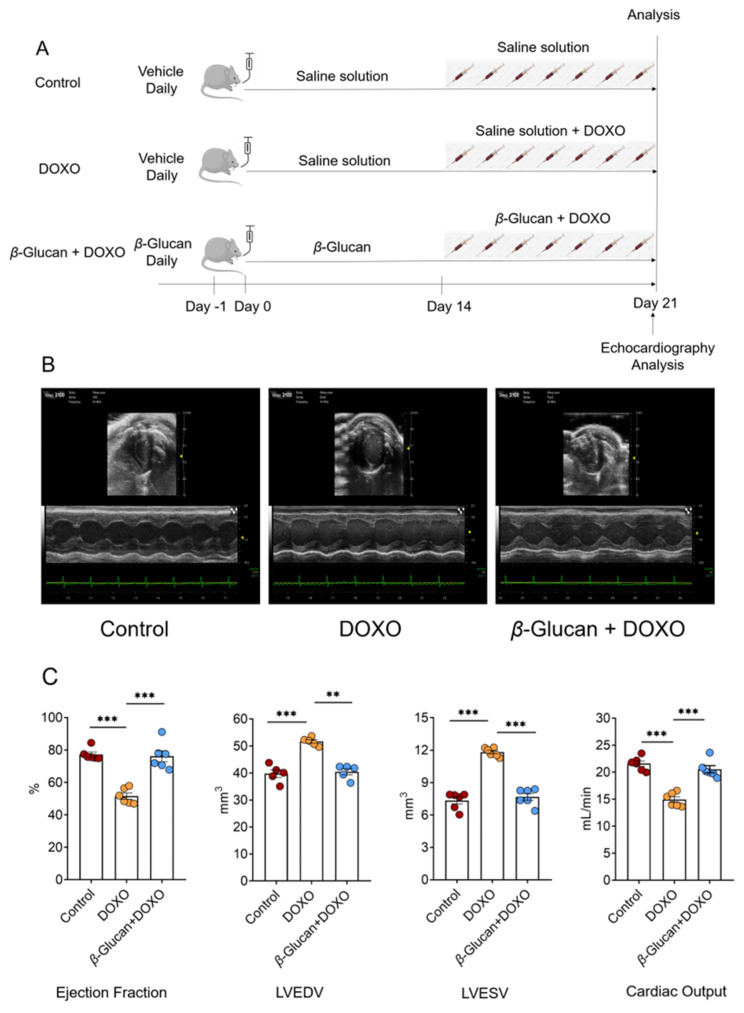
(**A**) Schematic protocol for mice treatments and echocardiography. C57/BL6J mice were randomly divided into three groups (*n* = 6 in each group). At day 0, mice in the *β*-glucan + DOXO groups were pre-treated with *β*-glucan daily for 21 days by oral gavage, while control and DOXO mice received vehicle. At day 15, DOXO and *β*-glucan + DOXO mice were injected with DOXO 1 h after daily pre-treatment with vehicle or *β*-glucan for the next 7 days, while control mice were treated with saline solution. At day 21, mice were sacrificed for ex vivo analysis. Heart function was monitored by echocardiography analysis at day 21. (**B**,**C**) *β*-glucan prevents left ventricular dilatation induced by DOXO. Top: sample M-mode short-axis echocardiographic images showing left ventricular dilatation induced by DOXO, and the protective effects of *β*-glucan in the *β*-glucan + DOXO group. Bottom: in mice treated with *β*-glucan + DOXO, left ventricular end-diastolic volume and left ventricular end-systolic volume are significantly smaller compared to DOXO group, and ejection fraction and cardiac output are significantly higher than that in the DOXO group. ** *p* < 0.01; *** *p* < 0.001.

Eight-week-old male C57BL/6J mice were provided by Beijing Vital River Laboratory Animal Technology Co., Ltd. The mice (*n* = 18) were randomly divided into 3 groups of 6 mice each. Control-treated mice (Control) group orally received an equivalent volume of placebo (saline) daily for 21 days. DOXO-treated mice group orally received an equivalent volume of placebo (saline) daily for 14 days, then a cumulative dose of 7 mg/kg DOXO (Cat#23214-92-8, Targetmol, MA, USA) via seven daily intraperitoneal injections (1 mg/kg/day each, DOXO group). DOXO plus *β*-glucan (Cat#9041-22-9, Shanghai Yuanye, Shanghai, China) treatment mice (*β*-glucan + DOXO) group, after 14 days of *β*-glucan administration (intragastrically at a dose of 500 mg/kg/day each), received co-administration of *β*-glucan (1h before administration of DOXO) and DOXO (at the same doses used in the DOXO group) (Figure 1A).

### 2.3. Echocardiography 

Using imaging system, Vevo 3100 High-Resolution in Vivo Micro-Imaging System (FUJIFILM VisualSonics, Toronto, ON, Canada), perform thoracic echocardiography on anesthetized mice. Left ventricle (LV) echocardiography is evaluated in parasternal long-axis and short-axis views at a frame rate of 233 Hz. End-diastolic and end-systolic are the phases corresponding to the T wave and R wave of the ECG, M-type LV end-diastolic dimensions (LVEDD) and LV end-systolic dimensions (LVESD) are the average of 3–5 heartbeats. LV M-mode detects LVEDD and LVESD at the papillary muscle level. Left ventricular ejection fraction was calculated as described previously (ejection fraction [%] = (LVIDd^3^ − LVIDs^3^)/LVIDd^3^; LVID, left ventricular diastolic; d, diastole; s, systole) [18]. All the studies and analysis were performed blinded to heart condition.

### 2.4. Immunohistological Analysis

Hearts was fixed overnight in 4% paraformaldehyde (pH 7.4), embedded in paraffin, and serially sectioned at a thickness of 5 μm. Sections were stained with hematoxylin and eosin (H&E) for routine histological examination with an optical microscope. In order to measure collagen deposits, selected sections were stained with Sirius Red. For each mouse, use ImageJ software to quantify three adjacent slices.

### 2.5. Serum Biochemical Index Detection

Whole blood samples were placed at room temperature for 2 h, then centrifuged at 2–8 °C at 3000 rpm for 15 min, and the supernatant was taken as serum. Serum aspartate aminotransferase (AST), creatine kinase (CK) and lactate dehydrogenase (LDH-L) were measured using the kits (Cat#S03040, S03024, S03034, Rayto, Shenzhen, China), serum lactate dehydrogenase isoenzyme 1 (LDH-1) and creatine Kinase Isoenzyme (CK-MB) were measured using the kits (Cat#C058-e, C060, Changchun Huili, Changchun, China). The specific method is as follows: referring to the instruction manual, using the double reagent method, and applying R1 and R2, respectively. Set the corresponding parameters of the corresponding indicators on the automatic biochemical analyzer, and use the rate method to detect the corresponding indicators of the serum samples under the main wavelength of 340 nm and the secondary wavelength of 405 nm. Serum glutathione peroxidase (GSH-PX), malondialdehyde (MDA) and Superoxide dismutase (SOD) were measured using the kits (Cat#CA005, A003-1, A001-1, NanJing JianCheng, Nanjing, China). The specific steps are as follows: according to the instructions of different reagent kits, add different reaction reagent kits, react according to the temperature and time specified by the reagent kit, use 1cm optical path cuvette, measure the OD value of each tube at the wavelengths of 412 nm, 532 nm and 550 nm, respectively. All above indicators are tested according to the manufacturer’s instructions. 

### 2.6. Measurements of ATP and ROS Content

ATP levels in cardiac tissue were detected using the Enhanced ATP Assay Kit (kit Cat#S0027, Beyotime, Shanghai, China) according to the manufacturer’s protocol. Briefly, the ATP working solution was added to the assay wells at room temperature, followed by the tissue lysis supernatant, and the RLU value was measured with a luminometer after mixing at least 2 s at room temperature. The effectiveness of the kit can refer to the relevant literature [19,20]. The fluorescent probe DCFH-DA (kit S0033S, Beyotime) was used to detect ROS in cardiac tissue according to the manufacturer’s instructions. DCFH-DA was added to the lysed tissue, incubated in a 37 °C incubator for 20 min, washed with PBS for 3 times, and quantitatively analyzed with a fluorescence microplate reader. For the effectiveness of the kit, please refer to the relevant literature [21,22].

### 2.7. Quantitative Real-Time PCR 

Trizol kit (Cat#CW0580S, Cwbio, Beijing, China) separates and extracts total RNA from mouse heart, and detects the concentration and purity of RNA with a spectrophotometer by Nanodrop 2000 (Thermo Scientific, Waltham, MA, USA). According to the manufacturer’s instructions and relevant literature reports [23,24], the experimental method is as follows: using HiScript III RT SuperMix for qPCR for reverse transcription (Cat#R323-01, Vazyme, Nanjing, China). Using Taq Pro Universal SYBR qPCR Master Mix (Cat#Q712-02, Vazyme) for quantitative PCR in accordance with the manufacturer’s instructions. The fold difference in gene expression was calculated using the 2^−ΔΔCt^ method and is presented relative to *Gapdh* mRNA. All reactions were performed in triplicate, and specificity was monitored using melting curve analysis [23,24,25].

### 2.8. Statistical Analysis

All statistical calculations were analyzed using GraphPad Prism 8 software, and all summary data are expressed as mean ± SEM. The student’s *t* test is used to compare two conditions, and one-way ANOVA with Bonferroni correction is used multiple comparisons. Probability values less than 0.05 were considered important.

## 3. Results

### 3.1. β-glucan Prevents DOXO-Induced Left Ventricular Dysfunction

We tested the protective effects of *β*-glucan on heart damage induced by DOXO (Figure 1A). A group of animals was treated with saline (control group) alone for 21 days. Another group of animals was treated with saline alone for 14 days, then the mice received a cumulative dose of 7 mg/kg DOXO via seven daily intraperitoneal injections of DOXO (DOXO group). The last group, after 14 days of pre-treatment with *β*-glucan alone, DOXO and *β*-glucan were administered together for 7 days (*β*-glucan + DOXO group). 

After 7 days of treatment with DOXO, transthoracic echocardiogram in vivo shows the LV dilation (Table 1): LV end-diastolic volume (LVEDV) is 51.69 ± 1.25 mm^3^ in DOXO group vs. 39.81 ± 2.62 mm^3^ in control group (*p* < 0.0001); LV end-systolic volume (LVESV) was 11.78 ± 0.35 mm^3^ in DOXO group vs. 7.23 ± 0.67 mm^3^ in control group (*p* < 0.0001); LV end-diastolic internal dimension (LVIDd) was 4.01 ± 0.13 mm in DOXO group vs. 3.46 ± 0.11 mm in control group (*p* < 0.0001). LV end-systole internal dimension (LVISd) was 2.84 ± 0.12 mm in DOXO group vs. 1.93 ± 0.16 mm in control group (*p* < 0.0001). Compared with the control group, the left ventricular ejection fraction of mice was significantly impaired after DOXO treatment (Figure 1B and Table 1).

Intriguingly, treatment with *β*-glucan resulted in a significant inhibition of all DOXO-induced effects (Figure 1B and Table 1). Indeed, mice from the *β*-glucan + DOXO group had significantly smaller left ventricles compared to DOXO group: LVEDV was 40.49 ± 2.12 mm^3^, LVESV was 7.92 ± 0.42 mm^3^, LVIDd was 3.29 ± 0.33 mm, and LVISd was 1.82 ± 0.36 mm (Figure 1B and Table 1). 

### 3.2. β-glucan Prevents DOXO-Induced Cardiac Remodeling and Injury

**Figure 2:**
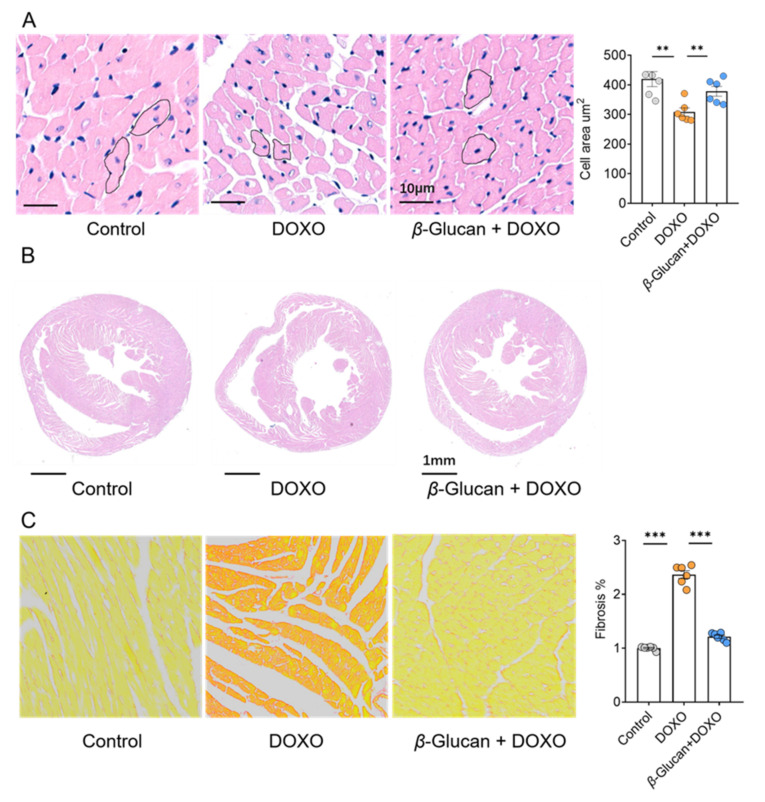
*β*-glucan protects heart from remodeling induced by DOXO. (**A**) *β*-glucan protects the heart from reduction of cardiomyocyte size induced by DOXO. (**B**) *β*-glucan protects the heart from DOXO-induced increases in cardiac volume. (**C**) *β*-glucan reduces interstitial fibrosis provoked by DOXO in the heart. *n* = 6 in each group. ** *p* < 0.01; *** *p* < 0.001.

Two important signs of DOXO-induced cardiotoxicity were further analyzed in the heart: cardiomyocyte size and cardiac fibrosis. *β*-glucan prevented the reduction in cardiomyocyte size induced by DOXO (Figure 2A). We found that treatment of these animals with DOXO increased ventricular volume, however, *β*-glucan ameliorated these effects (Figure 2B). Although DOXO injection induced moderate interstitial fibrosis, only minor interstitial fibrosis was detected after *β*-glucan treatment (Figure 2C).

### 3.3. β-glucan Blunts Myocardial Damage Induced by DOXO

**Figure 3:**
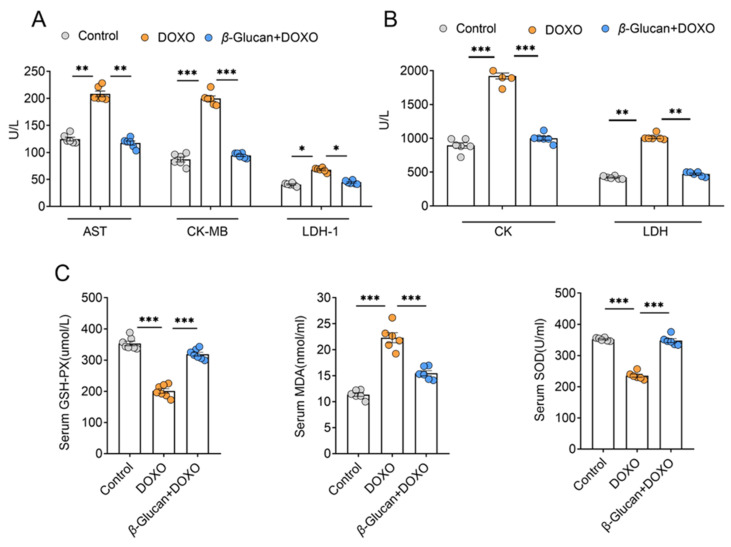
*β*-glucan plays an important role in DOXO-induced myocardial injury. (**A**,**B**) Serum AST, CK-MB, LDH-1, CK and LDH levels. (**C**) Serum GSH-PX, MDA, SOD levels. *n* = 6 in each group. * *p* < 0.05; ** *p* < 0.01; *** *p* < 0.001.

By detecting the levels of myocardial enzyme markers in the serum, we found LDH and its isoenzymes LDH-1, AST, CK and its isoenzymes CK-MB were all significantly increased after DOXO induction (Figure 3A,B). However, *β*-glucan treatment makes the expression levels of these markers similar to the control group, suggesting that the myocardial damage can be reduced by *β*-glucan (Figure 3A,B). Due to the induction of DOXO, the activity of antioxidant enzymes, GSH-PX, SOD in the oxidatively damaged serum is reduced, and *β*-glucan increases the activity of these enzymes (Figure 3C). The opposite results were observed with respect to DOXO-induced changes in serum levels of MDA, the most prevalent by-product of lipid peroxidation, *β*-glucan significantly reduced the level of MDA in the serum (Figure 3C).

### 3.4. β-glucan Improves the Reduction in Energy Production and the Increase in Oxidative Stress Caused by DOXO in Myocardial Tissue 

**Figure 4:**
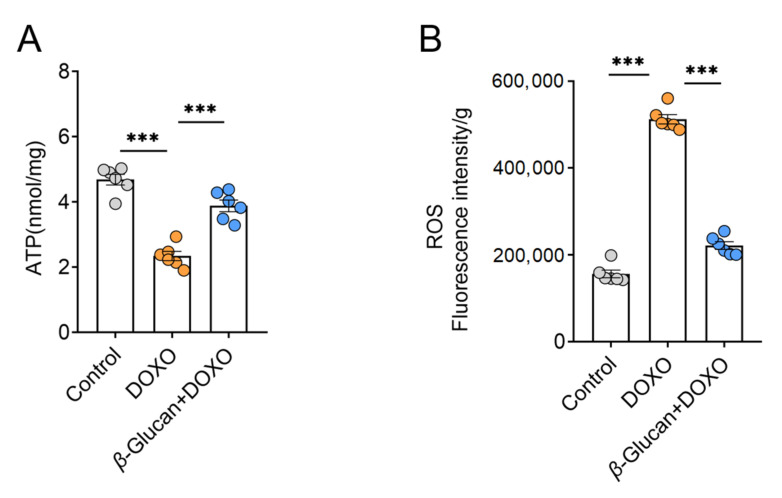
*β*-glucan plays a key role in improving mitochondrial function changes of cardiomyocytes induced by DOXO. (**A**) Measurement of ATP levels in heart tissue with or without *β*-glucan in DOXO-treated mice (*n* = 6 mice per group); (**B**) With or without *β*-glucan treatment, ROS levels in heart tissues were measured in DOXO-treated mice (*n* = 6 mice per group). *** *p* < 0.001.

Mitochondrial dysfunction has become a clear sign of DOXO-induced cardiotoxicity, and more and more evidence supports the key role of mitochondria in determining the fate of cardiomyocytes [26]. A mechanism suggests that mitochondria play a key role in the apoptotic pathway during DOXO-induced cardiotoxicity-ETC (Electron Transport Respiratory Chain) interruption with ATP production, the release of proteins that trigger the activation of the caspase protease family, and changes in the redox potential [27,28,29]. At the same time, this process of producing ATP through the respiratory chain leads to the production of ROS as a metabolic by-product [30]. Our results show that *β*-glucan reversed the DOXO-induced decrease in the ATP synthesis (Figure 4A), as well as the DOXO-induced increase in ROS production (Figure 4B), suggesting that *β*-glucan may protect mitochondrial function. 

### 3.5. β-glucan Improves Mitochondrial Function Caused by DOXO and Reduces Heart Damage 

**Figure 5:**
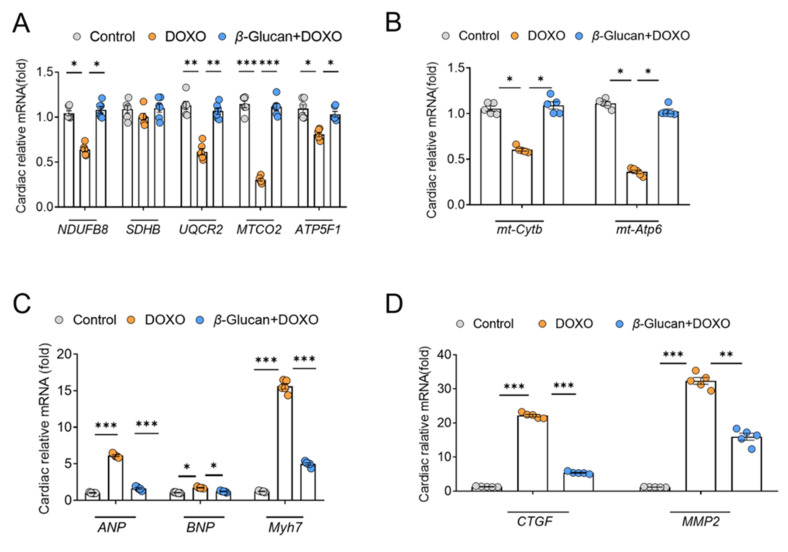
*β*-glucan reduces DOXO-induced cardiotoxicity by improving the mitochondrial function of the mouse heart. (**A**) mRNA expression levels of mitochondrial complex-related genes are decreased in mice treated with DOXO, while they are increased in the *β*-glucan + DOXO group. (**B**) *β*-glucan restores mRNA expression levels related to mitochondrial function after DOXO induction. (**C**,**D**) Heart function is impaired after DOXO induction, but *β*-glucan can repair this damage. *n* = 6 in each group. * *p* < 0.05; ** *p* < 0.01; *** *p* < 0.001.

From the results in Figure 4, it can be seen that the ROS in the heart tissue induced by DOXO was significantly increased. The complex of the mitochondrial respiratory chain is one of the main providers of ROS in most cells [31]. *NDUFB*8 [32], *SDHB* [33], *UQCR2* [34], *MTCO2* [35] and *ATP5F1* [36] are the marker genes of mitochondrial complex I-V, respectively. Therefore, we detected the mRNA expression level of these mitochondrial complex related genes in heart tissue after DOXO induction. After DOXO induction, except for the mitochondrial complex II marker *UQCR2*, the mRNA levels of other complex marker genes were significantly decreased (Figure 5A), which indicates that DOXO reduced the activity of mitochondrial respiratory chain complex, leading to increased ROS production.

Then we detected two genes encoded by mitochondria, mitochondrial ATPase6 (*mt-ATP6*) and mitochondrial cytochrome b (*mt-Cytb*). After DOXO induction, their mRNA levels were down-regulated, while *β*-glucan can restore the mRNA expression of these genes (Figure 5B). These results indicate that DOXO does indeed decrease mitochondrial function by reducing the expression of mitochondrial genes. Fortunately, *β*-glucan can improve the dysfunction of cardiac mitochondria induced by DOXO (Figure 5A,B).

In addition, we detected the mRNA expression of the hallmark genes of heart function. Compared to the control group, a significant increased expression of *ANP*, *BNP* and *Myh7*, as markers of heart dysfunction, was found in the DOXO group (Figure 5C). Interestingly, oral treatment with *β*-glucan can prevent such increase compared to the DOXO group (Figure 5C). Not surprisingly, compared with the control group, the increase in the expression of *ANP*, *BNP* and *Myh7* was also accompanied by a significant increase in the expression of *CTGF* and *MMP-2* genes in the DOXO group, confirming the activation of cardiac remodeling (Figure 5D). Co-treatment with *β*-glucan significantly reduced these mRNAs compared to the DOXO group (Figure 5D). 

## 4. Discussion

Here, we report that DOXO administration can lead to left ventricular dysfunction, and treatment with *β*-glucan significantly inhibits all DOXO-induced effects. In fact, the incidence of cardiotoxicity caused by DOXO is not optimistic. A study reported that of the 4018 patients treated with DOXO, 2.2% had symptoms of heart failure [37]. DOXO elevates the content of oxygen free radicals in the heart, which can cause myocardial damage and even heart failure in severe cases [38,39]. Ischemia/reperfusion, atherosclerosis and other heart-related diseases are all related to the increase of ROS content. DOXO can break the dynamic balance between antioxidant enzymes and ROS in the cell, indirectly cause cell apoptosis and destruction of Ca^2+^ homeostasis [31,40,41,42,43]. Since cardiomyocytes have low levels of antioxidant enzymes (such as GSH-PX and SOD), these cells are more susceptible to oxidative damage. In addition, DOXO may induce mitochondrial function damage which leads to insufficient cell energy supply and impaired mitochondrial respiratory chain system, ultimately triggering apoptosis and necrosis. 

The natural *β*-glucan has received attention for many years due to its physical and chemical properties. *β*-glucan has a variety of physiological and biochemical functions, such as improving lipid metabolism, anti-tumor, antibacterial, antioxidant, anti-inflammatory, etc. Studies have reported that *β*-glucan can effectively reduce oxidative stress parameters, scavenge free radicals and enhance Fe^2+^ chelating ability [44,45]. Oatmeal and barley contain 4–10%*w*/*w β*-glucan [46]. Daily consumption of oatmeal and barley can significantly reduce low-density lipoprotein cholesterol and blood cholesterol [47,48,49]. Studies have reported that continuous consumption of natural oatmeal supplemented with 6 g/day of *β*-glucan has a good effect on glycemic control and variability in adolescents with type 1 diabetes [50]. Pretreatment of high-risk surgical patients with intravenous *β*-glucan reduces the incidence of infection and the need for antibiotics [51]. It may be possible to modulate immune function intake by increasing dietary *β*-glucan, for example by developing functional foods [11]. Therefore, *β*-glucan is a beneficial compound for animal and human health. However, there is no relevant literature report whether *β*-glucan can improve the oxidative stress damage of the heart induced by DOXO. In our study, analysis of cardiac fibrosis showed that DOXO-induced cardiac interstitial fibrosis was significantly increased, which was offset by co-administration of *β*-glucan. Similarly, *β*-glucan prevented the DOXO-induced reduction in cardiomyocyte size. From the biochemical indicators detected in the isolated serum, it is known that the DOXO-induced increase in myocardial enzymes, including LDH, LDH-1, AST, CK and CK-MB, show that cardiomyocytes are damaged, and *β*-glucan can reduce the damage of cardiomyocytes. In addition, *β*-glucan can also alleviate the increase in serum MDA concentration caused by DOXO, and enhance the activity of SOD and GSH-PX. In other words, *β*-glucan reduces the oxidative damage induced by DOXO. It has been reported in the literature that *β*-glucan treatment can prevent acetaminophen-induced liver toxicity [17], while our study shows that *β*-glucan can improve DOXO-induced cardiotoxicity and the effect is more significant. This may be due to the longer gavage period and larger dose of *β*-glucan in our study. In addition to *β*-glucan acting as an antioxidant to reduce DOXO-induced oxidative damage, it is also possible that DOXO and *β*-glucan metabolites form aggregates, resulting in the inactivation of DOXO and ineffectiveness. Together, we aimed to explore the mechanism of *β*-glucan to improve DOXO induced cardiotoxicity, and found that *β*-glucan can improve cardiotoxicity by reducing ROS levels and increasing ATP production, thereby reducing oxidative stress and improving mitochondrial function.

In conclusion, from a clinical point of view, our findings suggest that *β*-glucan is well suited for relieving DOXO-induced heart failure. From an antioxidant perspective, we found that *β*-glucan can reduce DOXO-induced oxidative stress. However, we do not rule out that the metabolites of *β*-glucan may form aggregates with DOXO, leading to the loss of DOXO activity and protection of the heart. Therefore, more studies are needed to better define the potential of *β*-glucan in cardiology and further explore the relevant molecular mechanisms to elucidate the potential clinical implications of this therapeutic strategy. 

## Figures and Tables

**Table 1:** Echocardiographic parameters after *β*-glucan treatment.

	Control (*n* = 6)	DOXO (*n* = 6)	*β*-Glucan + DOXO (*n* = 6)
	Mean ± SEM	Mean ± SEM	Mean ± SEM
EF (%)	77.27 ± 3.33	51.55 ± 4.31 ***	76.21 ± 7.61 ^###^
CO (mL/min)	21.59 ± 1.16	14.94 ± 1.17 ***	20.54 ± 1.49 ^###^
LVIDd (mm)	3.46 ± 0.11	4.01 ± 0.13 ***	3.29 ± 0.33 ^##^
LVISd (mm)	1.93 ± 0.16	2.84 ± 0.12 ***	1.82 ± 0.36 ^###^
FS (%)	45.27 ± 3.43	26.04 ± 2.78 ***	41.36 ± 3.06 ^###^
LV Mass (mg)	41.58 ± 2.32	24.47 ± 1.30 ***	43.11 ± 2.40 ^###^
LVAWd (mm)	0.41 ± 0.03	0.47 ± 0.06	0.50 ± 0.06
LVAWs (mm)	0.37 ± 0.02	0.37 ± 0.06	0.35 ± 0.03
LVPWd (mm)	0.62 ± 0.05	0.63 ± 0.06	0.59 ± 0.04
LVPWs (mm)	0.99 ± 0.05	0.79 ± 0.06 **	1.00 ± 0.04 ^###^
SV (μL)	45.79 ± 1.64	30.96 ± 2.56 ***	44.44 ± 2.58 ^###^
LVEDV (mm^3^)	39.81 ± 2.62	51.69 ± 1.25 ***	40.49 ± 2.12 ^###^
LVESV (mm^3^)	7.23 ± 0.67	11.78 ± 0.35 ***	7.92 ± 0.42 ^###^

EF, ejection fraction; CO, cardiac output; LVID, left ventricular diastolic; LVIS, left ventricular systolic; FS, fractional shortening; LV Mass, left ventricular mass; LVAW, left ventricular anterior wall thickness; LVPW, left ventricular posterior wall thickness; SV, stroke volume; LVEDV, left ventricular end-diastolic volume; LVESV, left ventricular end-systolic volume; d, diastole; s, systole. ** *p* < 0.01, *** *p* < 0.001 (DOXO vs. Control); ^##^ *p* < 0.01, ^###^ *p* < 0.001 (*β*-glucan + DOXO vs. DOXO).

## Data Availability

Not applicable.

## References

[1] Singal P.K., Iliskovic N. (1998). Doxorubicin-induced cardiomyopathy. N. Engl. J. Med..

[2] Syahputra R.A., Harahap U., Dalimunthe A., Pandapotan M., Satria D. (2021). Protective effect of Vernonia amygdalina Delile against doxorubicin-induced cardiotoxicity. Heliyon.

[3] Zafar A., Rizvi A., Ahmad I., Ahmad M. (2014). Habitat of in vivo transformation influences the levels of free radical scavengers in Clinostomum complanatum: Implications for free radical scavenger based vaccines against trematode infections. PLoS ONE.

[4] Hameed A., Hussain S.A., Yang J., Ijaz M.U., Liu Q., Suleria H.A.R., Song Y. (2017). Antioxidants Potential of the Filamentous Fungi (*Mucor circinelloides*). Nutrients.

[5] Huang L.J., Chen S.R., Yuan C.M., Gu W., Guo B.J., Wang Y.T., Wang Y., Hao X.J. (2017). C(21)-steroidal pregnane sapogenins and their derivatives as anti-inflammatory agents. Bioorg. Med. Chem..

[6] Hasinoff B.B., Herman E.H. (2007). Dexrazoxane: How it works in cardiac and tumor cells. Is it a prodrug or is it a drug?. Cardiovasc. Toxicol..

[7] Ichikawa Y., Ghanefar M., Bayeva M., Wu R., Khechaduri A., Naga Prasad S.V., Mutharasan R.K., Naik T.J., Ardehali H. (2014). Cardiotoxicity of doxorubicin is mediated through mitochondrial iron accumulation. J. Clin. Investig..

[8] Feng S.-S., Chien S. (2003). Chemotherapeutic engineering: Application and further development of chemical engineering principles for chemotherapy of cancer and other diseases. Chem. Eng. Sci..

[9] Abraham S.A., Waterhouse D.N., Mayer L.D., Cullis P.R., Madden T.D., Bally M.B. (2005). The liposomal formulation of doxorubicin. Methods Enzym..

[10] Zhu F., Du B., Bian Z., Xu B. (2015). Beta-glucans from edible and medicinal mushrooms: Characteristics, physicochemical and biological activities. J. Food Compos. Anal..

[11] Volman J.J., Ramakers J.D., Plat J. (2008). Dietary modulation of immune function by beta-glucans. Physiol. Behav..

[12] Ciecierska A., Drywień M.E., Hamulka J., Sadkowski T. (2019). Nutraceutical functions of beta-glucans in human nutrition. Rocz. Panstw. Zakl. Hig..

[13] Meijer K., de Vos P., Priebe M.G. (2010). Butyrate and other short-chain fatty acids as modulators of immunity: What relevance for health?. Curr. Opin. Clin. Nutr. Metab. Care.

[14] Hague A., Elder D.J., Hicks D.J., Paraskeva C. (1995). Apoptosis in colorectal tumour cells: Induction by the short chain fatty acids butyrate, propionate and acetate and by the bile salt deoxycholate. Int. J. Cancer.

[15] Kimura I., Ozawa K., Inoue D., Imamura T., Kimura K., Maeda T., Terasawa K., Kashihara D., Hirano K., Tani T. (2013). The gut microbiota suppresses insulin-mediated fat accumulation via the short-chain fatty acid receptor GPR43. Nat. Commun..

[16] Chan G.C., Chan W.K., Sze D.M. (2009). The effects of beta-glucan on human immune and cancer cells. J. Hematol. Oncol..

[17] Toklu H.Z., Sehirli A.O., Velioğlu-Oğünç A., Cetinel S., Sener G. (2006). Acetaminophen-induced toxicity is prevented by beta-D-glucan treatment in mice. Eur. J. Pharm..

[18] Russo M., Guida F., Paparo L., Trinchese G., Aitoro R., Avagliano C., Fiordelisi A., Napolitano F., Mercurio V., Sala V. (2019). The novel butyrate derivative phenylalanine-butyramide protects from doxorubicin-induced cardiotoxicity. Eur. J. Heart Fail..

[19] Li Y., Ye Z., Lai W., Rao J., Huang W., Zhang X., Yao Z., Lou T. (2017). Activation of Sirtuin 3 by Silybin Attenuates Mitochondrial Dysfunction in Cisplatin-induced Acute Kidney Injury. Front. Pharm..

[20] Zhou W., Tian D., He J., Zhang L., Tang X., Zhang L., Wang Y., Li L., Zhao J., Yuan X. (2017). Exposure scenario: Another important factor determining the toxic effects of PM2.5 and possible mechanisms involved. Env. Pollut..

[21] Liu M.X., Jin L., Sun S.J., Liu P., Feng X., Cheng Z.L., Liu W.R., Guan K.L., Shi Y.H., Yuan H.X. (2018). Metabolic reprogramming by PCK1 promotes TCA cataplerosis, oxidative stress and apoptosis in liver cancer cells and suppresses hepatocellular carcinoma. Oncogene.

[22] Yuan S.X., Li J.L., Xu X.K., Chen W., Chen C., Kuang K.Q., Wang F.Y., Wang K., Li F.C. (2018). Underlying mechanism of the photodynamic activity of hematoporphyrin-induced apoptosis in U87 glioma cells. Int. J. Mol. Med..

[23] Fang X., Wang H., Han D., Xie E., Yang X., Wei J., Gu S., Gao F., Zhu N., Yin X. (2019). Ferroptosis as a target for protection against cardiomyopathy. Proc. Natl. Acad. Sci. USA.

[24] Kory N., Uit de Bos J., van der Rijt S., Jankovic N., Güra M., Arp N., Pena I.A., Prakash G., Chan S.H., Kunchok T. (2020). MCART1/SLC25A51 is required for mitocho.ondrial NAD transport. Sci. Adv..

[25] Mancina R.M., Sasidharan K., Lindblom A., Wei Y., Ciociola E., Jamialahmadi O., Pingitore P., Andréasson A.C., Pellegrini G., Baselli G. (2022). PSD3 downregulation confers protection against fatty liver disease. Nat. Metab..

[26] Govender J., Loos B., Marais E., Engelbrecht A.M. (2014). Mitochondrial catastrophe during doxorubicin-induced cardiotoxicity: A review of the protective role of melatonin. J. Pineal. Res..

[27] Tokarska-Schlattner M., Zaugg M., Zuppinger C., Wallimann T., Schlattner U. (2006). New insights into doxorubicin-induced cardiotoxicity: The critical role of cellular energetics. J. Mol. Cell Cardiol..

[28] Green D.R., Reed J.C. (1998). Mitochondria and apoptosis. Science.

[29] Sainz R.M., Mayo J.C., Rodriguez C., Tan D.X., Lopez-Burillo S., Reiter R.J. (2003). Melatonin and cell death: Differential actions on apoptosis in normal and cancer cells. Cell Mol. Life Sci..

[30] Chen Y.R., Zweier J.L. (2014). Cardiac mitochondria and reactive oxygen species generation. Circ. Res..

[31] Brand M.D. (2016). Mitochondrial generation of superoxide and hydrogen peroxide as the source of mitochondrial redox signaling. Free Radic. Biol. Med..

[32] Piekutowska-Abramczuk D., Assouline Z., Mataković L., Feichtinger R.G., Koňařiková E., Jurkiewicz E., Stawiński P., Gusic M., Koller A., Pollak A. (2018). NDUFB8 Mutations Cause Mitochondrial Complex I Deficiency in Individuals with Leigh-like Encephalomyopathy. Am. J. Hum. Genet..

[33] Lemarie A., Grimm S. (2009). Mutations in the heme b-binding residue of SDHC inhibit assembly of respiratory chain complex II in mammalian cells. Mitochondrion.

[34] Barros M.H., McStay G.P. (2020). Modular biogenesis of mitochondrial respiratory complexes. Mitochondrion.

[35] Rak M., Bénit P., Chrétien D., Bouchereau J., Schiff M., El-Khoury R., Tzagoloff A., Rustin P. (2016). Mitochondrial cytochrome c oxidase deficiency. Clin. Sci..

[36] Brüggemann M., Gromes A., Poss M., Schmidt D., Klümper N., Tolkach Y., Dietrich D., Kristiansen G., Müller S.C., Ellinger J. (2017). Systematic Analysis of the Expression of the Mitochondrial ATP Synthase (Complex V) Subunits in Clear Cell Renal Cell Carcinoma. Transl. Oncol..

[37] Von Hoff D.D., Layard M.W., Basa P., Davis H.L., Von Hoff A.L., Rozencweig M., Muggia F.M. (1979). Risk factors for doxorubicin-induced congestive heart failure. Ann. Intern. Med..

[38] Tacar O., Sriamornsak P., Dass C.R. (2013). Doxorubicin: An update on anticancer molecular action, toxicity and novel drug delivery systems. J. Pharm. Pharm..

[39] de Wolf F.A. (1991). Binding of doxorubicin to cardiolipin as compared to other anionic phospholipids--an evaluation of electrostatic effects. Biosci. Rep..

[40] Petronilli V., Costantini P., Scorrano L., Colonna R., Passamonti S., Bernardi P. (1994). The voltage sensor of the mitochondrial permeability transition pore is tuned by the oxidation-reduction state of vicinal thiols. Increase of the gating potential by oxidants and its reversal by reducing agents. J. Biol. Chem..

[41] Schieber M., Chandel N.S. (2014). ROS function in redox signaling and oxidative stress. Curr. Biol..

[42] Harman D. (1956). Aging: A theory based on free radical and radiation chemistry. J. Gerontol..

[43] Chouchani E.T., Pell V.R., Gaude E., Aksentijević D., Sundier S.Y., Robb E.L., Logan A., Nadtochiy S.M., Ord E.N.J., Smith A.C. (2014). Ischaemic accumulation of succinate controls reperfusion injury through mitochondrial ROS. Nature.

[44] Liu W., Wang H., Pang X., Yao W., Gao X. (2010). Characterization and antioxidant activity of two low-molecular-weight polysaccharides purified from the fruiting bodies of Ganoderma lucidum. Int. J. Biol. Macromol..

[45] Lei N., Wang M., Zhang L., Xiao S., Fei C., Wang X., Zhang K., Zheng W., Wang C., Yang R. (2015). Effects of Low Molecular Weight Yeast β-Glucan on Antioxidant and Immunological Activities in Mice. Int. J. Mol. Sci..

[46] Burton R.A., Fincher G.B. (2012). Current challenges in cell wall biology in the cereals and grasses. Front. Plant. Sci.

[47] Whitehead A., Beck E.J., Tosh S., Wolever T.M. (2014). Cholesterol-lowering effects of oat β-glucan: A meta-analysis of randomized controlled trials. Am. J. Clin. Nutr..

[48] Wolever T.M., Tosh S.M., Gibbs A.L., Brand-Miller J., Duncan A.M., Hart V., Lamarche B., Thomson B.A., Duss R., Wood P.J. (2010). Physicochemical properties of oat β-glucan influence its ability to reduce serum LDL cholesterol in humans: A randomized clinical trial. Am. J. Clin. Nutr..

[49] Tiwari U., Cummins E. (2011). Meta-analysis of the effect of β-glucan intake on blood cholesterol and glucose levels. Nutrition.

[50] Bozbulut R., Şanlıer N., Döğer E., Bideci A., Çamurdan O., Cinaz P. (2020). The effect of beta-glucan supplementation on glycemic control and variability in adolescents with type 1 diabetes mellitus. Diabetes Res. Clin. Pract..

[51] Babineau T.J., Hackford A., Kenler A., Bistrian B., Forse R.A., Fairchild P.G., Heard S., Keroack M., Caushaj P., Benotti P. (1994). A phase II multicenter, double-blind, randomized, placebo-controlled study of three dosages of an immunomodulator (PGG-glucan) in high-risk surgical patients. Arch. Surg..

